# Resistance to Streptozotocin-Induced Autoimmune Diabetes in Absence of Complement C3: Myeloid-Derived Suppressor Cells Play a Role

**DOI:** 10.1371/journal.pone.0066334

**Published:** 2013-06-18

**Authors:** Xiaogang Gao, Huanhai Liu, Bin He, Zhiren Fu

**Affiliations:** 1 Department of Organ Transplantation, Shanghai ChangZheng Hospital, Second Military Medical University, Shanghai, P. R. China; 2 Department of Otolaryngology-Head and Neck Surgery, Shanghai ChangZheng Hospital, Second Military Medical University, Shanghai, P. R. China; 3 Department of Anesthesiology and Critical Care Medicine, Xinhua Hospital, School of Medicine, Shanghai Jiaotong University, Shanghai, P. R. China; University of Michigan Medical School, United States of America

## Abstract

The contribution of complement to the development of autoimmune diabetes has been proposed recently. The underlying mechanisms, however, remain poorly understood. We hypothesize that myeloid-derived suppressor cells (MDSC), which act as regulators in autoimmunity, play a role in resistance to diabetes in absence of complement C3. Indeed, MDSC number was increased significantly in STZ-treated C3−/− mice. These cells highly expressed arginase I and inducible nitric oxide synthase (iNOS). Importantly, depletion of MDSC led to the occurrence of overt diabetes in C3−/− mice after STZ. Furthermore, C3−/− MDSC actively suppressed diabetogenic T cell proliferation and prevented/delayed the development of diabetes in arginase and/or iNOS-dependent manner. Both Tregs and transforming growth factor-β (TGF-β) are crucial for MDSC induction in STZ-treated C3−/− mice as depletion of Tregs or blocking TGF-β bioactivity dramatically decreased MDSC number. These findings indicate that MDSC are implicated in resistance to STZ-induced diabetes in the absence of complement C3, which may be helpful for understanding of mechanisms underlying preventive effects of complement deficiency on autoimmune diseases.

## Introduction

The complement system, consisting of ∼30 soluble- and membrane-bound proteins, is one of central arms of innate immunity that are critical to host defense as well as the development of adaptive immunity [1]. Complement proteins interact with one another in one of three different sequential activation cascades known as the classical, alternative, and lectin pathways. Eventually, all three pathways converge, with complement protein C3 and C5, into one terminal cascade that leads to formation of the membrane attack complex [2]. Uncontrolled complement activation can lead to tissue inflammation or damage, which occurs in many immune-complex-mediated diseases such as rheumatoid arthritis, asthma, liver diseases, and renal diseases [3]–[6].

Recently, increasing evidence has shown that complement activation also has a positive effect on T-cell-mediated autoimmunity [7]–[9]. Administration of complement inhibitor FUT-175 potently suppressed autoreactive T cell response and prevented against the development of disease, in the experimental autoimmune encephalomyelitis model [8]. Furthermore, lack of the cell surface C3/C5 convertase inhibitor decay-accelerating factor (DAF) led to robust autoimmune response in attacked organs, with characteristics of accumulation of myelin-destructive IFN-γ^+^ and IL-17^+^ T cells [10]. The recent work from our lab and others has proposed the contribution of complement to the development of autoimmune diabetes [9], [11]. In absence of C3, streptozotocin-induced autoimmune diabetes failed to be established, which is characterized by loss of histologic insulitis [9]. The tolerance of T-cell reactivity to islets is associated with expansion of TGF-β-producing Tregs [11]. Whereas, whether other immune cell populations with immunosuppressive function are involved remains unknown.

Myeloid-derived suppressor cells (MDSC), with hallmarks of expressing Gr-1 and CD11b molecules, accumulate in large numbers during many pathological conditions, including cancer, infectious diseases, trauma, and autoimmunity [12]–[14]. MDSC are characterized by their potent ability to suppress different aspects of immune responses, especially T-cell proliferation and cytokine production. Several lines of studies have implicated up-regulation of arginase, NO, and reactive oxygen species as the major factors responsible for the immune suppressive activity of MDSC [15], [16]. Intriguingly, a protective role of MDSC in autoimmune diabetes has been proposed recently [17]. Transfer of MDSC significantly reduced the incidence of diabetes in recipients receiving diabetogenic CD4 T cells. This effect of MDSC might be mediated by inducing anergy in autoreacitve T cells and the development of Tregs. In the present study, we hypothesize that MDSC as key regulator for autoimmune response to insulin-producing cells redirect destructive T cells into tolerogenic actors thereby block the development of diabetes.

## Materials and Methods

### Mice

C3−/− and C3+/+ mice were purchased from Jackson Laboratory, bred and maintained on C57BL/6J in the animal facilities under specific pathogen-free conditions. Care, use and treatment of mice in this study were in strict agreement with international guidelines for the care and use of laboratory animals and approved by Animal Ethics Committee of Second Military Medical University.

### Induction of Diabetes by STZ

Mice were injected intraperitoneally for 5 consecutive days with 50 mg/kg body weight of STZ (Sigma Aldrich, St. Louis, MO) in 0.1 mol/L citrate buffer (pH 4.5) to induce diabetes. Blood glucose levels were monitored by testing blood glucose weekly using test strips (Abbott laboratories, USA). Mice with a blood glucose measurement of greater than 300 mg/dl for 2 consecutive weeks were considered diabetic.

### Assays for Cytokine Detection

The plasma samples were collected routinely. TGF-β level was determined by enzyme-linked immunosorbent assay (ELISA) according to manufacturer’s instructions. For insulin, serum was collected 3^rd^ week after STZ injection and insulin contents were determined by corresponding ELISA kit. The TGF-β ELISA kit was purchased from R&D Systems. Insulin ELISA kit was purchased Alpco.

### L-arginine Measurement

L-Arg concentration in tissue culture medium was measured by high-performance liquid chromatography with electron capture detection using an ESA-CoulArray Model 540 (ESA Inc., Chelmsford, MA) with an 80×3.2 column with 120A pore size. Briefly, supernatants were deproteinized in methanol. After centrifugation at 6,000×*g* for 10 min at 4°C, the supernatant was derivatized with 0.2 M *O*-phtaldialdehyde containing 7 mM *β*-mercaptoethanol. Fifty microliters of the sample were injected into the column. The retention time for L-Arg was 10.2 min. Standards for L-Arg were prepared in methanol.

### Flow Cytometry

Antibodies used for flow cytometry analysis are as follows: FITC-labeled anti-murine Gr-1 (RB6-8C5), Ly-6G (1A8) were purchased from *e*Biosciences (USA). PE-labeled anti-murine CD11b (M1/70), Ly-6C (HK1.4) and APC-labeled anti-murine CD11b (M1/70) were purchased from Biolegend (USA). For blood samples, whole blood was treated with FACS lysing solution (BD Biosciences) after immunofluorescence staining. Cell were stained in PBS with 2% heat-inactivated FCS and 0.2% sodium azide, and fixed using PBS with 1% paraformaldehyde. Data collection and analysis were performed on a FACS Calibur flow cytometry using CellQuest software (Becton Dickinson, USA).

### Antibody Depletion

Depletion of Gr-1^+^ cells was performed by injecting i.p. injection anti-Gr-1 antibodies (RB6-8C5; 250 µg) or control Isotype (rat IgG) twice a week until mice were sacrificed. >80% of MDSC were eliminated confirmed by flow cytometry. Depletion of Tregs was performed as previously described [11]. Briefly, anti-CD25 mAbs (PC61, BD Pharmingen) or isotype IgG was injected i.p. at the dose of 250 µg/mouse. The interval between the first and second injection was 3 days, thereafter injection once every 5 days until mice were sacrificed. The efficiency of elimination of Tregs was confirmed by flow cytometry.

### In vitro Coculture Experiments

Splenic CD4^+^ T cells were purified from STZ-treated diabetic WT mice using CD4^+^ magnetic microbeads, according to the manufacturer’s instructions (Miltenyi Biotec). MDSC (Gr-1^+^CD11b^+^) from spleen of C3+/+ or C3−/− STZ-treated mice were sorted by FACS Aria II (Becton Dickinson, USA) (purity>95%). CD4^+^ T cells or MDSC were seeded, alone or in coculture (at indicated ratio) in flat-bottomed 96-well microplates (2×10^4^ cells to 1×10^5^ cells/well; total volume of 200 µl/well). Cells were stimulated with immobilized CD3-specific antibody (0.5 µg/ml) and irradiated APC. When necessary, inhibitors of arginase (ASE) and/or iNOS included *N*-hydroxy-L-arginine (NOHA; 100 µM), an inhibitor of iNOS and ASE (Calbiochem, San Diego, CA), the specific ASE inhibitor *N*-hydroxy-nor-aiginine (nor-NOHA; 50 µM), and the NOS inhibitor L-*N*-iminoethyllysine (L-NIL, 5 µg/ml) were added to the cocultures. After 48 h in culture at 37°C with 5% CO_2_, 1 µCi (^3^H)thymidine was added to each well, and cells were harvested 12 h later. Proliferation was detected using a 1450 Microbeta liquid scintillation counter (PerkinElmer, Boston, MA).

### Neutralization of TGF-β in vivo

To assess the role of TGF-β in vivo, some mice received antibody to TGF-β (2G.7; R&D Systems) or isotype IgG. 1 mg was injected intraperitoneally on days 3, 5, 8, 10, 12 and 15 after STZ administration.

### Adoptive Transfer

Purified MDSC from the spleen of C3−/− STZ-treated mice or naïve wild-type mice were given i.v. into the tail veins of C3+/+ diabetic mice followed by STZ administration at indicated number. In some settings, mice received i.p. administration of arginase/iNOS inhibitors NOHA (150 mg/kg) simultaneously. Recipients were tested every week for diabetes and diagnosed as described above.

### Histopathology

Mice were sacrificed by cervical dislocation, and the pancreas was immediately removed. Each pancreas was fixed with 10% buffered formalin, embedded in paraffin, sectioned at 4.5 µm, and stained with hematoxylin and eosin. Insulitis grade was determined as follows: 0, normal islet; 1, mononuclear infiltration, largely in the periphery, in <25% of the islet; 2, 25–50% of the islets showing mononuclear infiltration; 3, >50% of the islet showing mononuclear infiltration; 4, small, retracted islet with few mononuclear cells.

### Quantitative Gene Expression

Total RNA was extracted TRIzol Reagent. cDNA was synthesized with SuperScript II reverse transcriptase (Invitrogen, Carlsbad, CA). The primers for arginase-1: (forward) 5′-CAC GGC AGT GGC TTT AAC CT-3′, (reverse) 5′-TGG CGC ATT CAC AGT CAC TT-3′; iNOS: (forward) 5′-TGG CCA CCT TGT TCA GCT ACG-3′, (reverse) 5′-GCC AAG GCC AAA CAC AGC ATA C-3′; IL-1β: (forward) 5′-AGG CCA CAG GTA TTT TGT C-3′, (reverse) 5′-GCC CAT CCT CTG TGA CTC A-3′; IL-6: (forward) 5′-CCA CTT CAC AAG TCG GAG GCT TA -3′, (reverse) 5′-GCA AGT GCA TCA TCG TTG TTC ATA C-3′; TNF-α: (forward) 5′-CTG GGA GTA GAC AAG GTA CAA CCC A-3′, (reverse) 5′-ATT CGA GTG ACA AGC CTG TAG CCC A-3′. The mRNAs were measured using Applied Biosystems 7500 Fast PCR System in duplicate, and normalized to 18 s mRNA.

### Statistical Analysis

Kaplan-Meier method was used to compare diabetes incidence. Student *t*-test was used to compare mean values. Values of *P*<0.05 were considered significant.

## Results

### C3 deficiency Confers Resistance to STZ-induced Diabetes and Prevents Leukocyte Infiltrates in Islets

**Figure 1:**
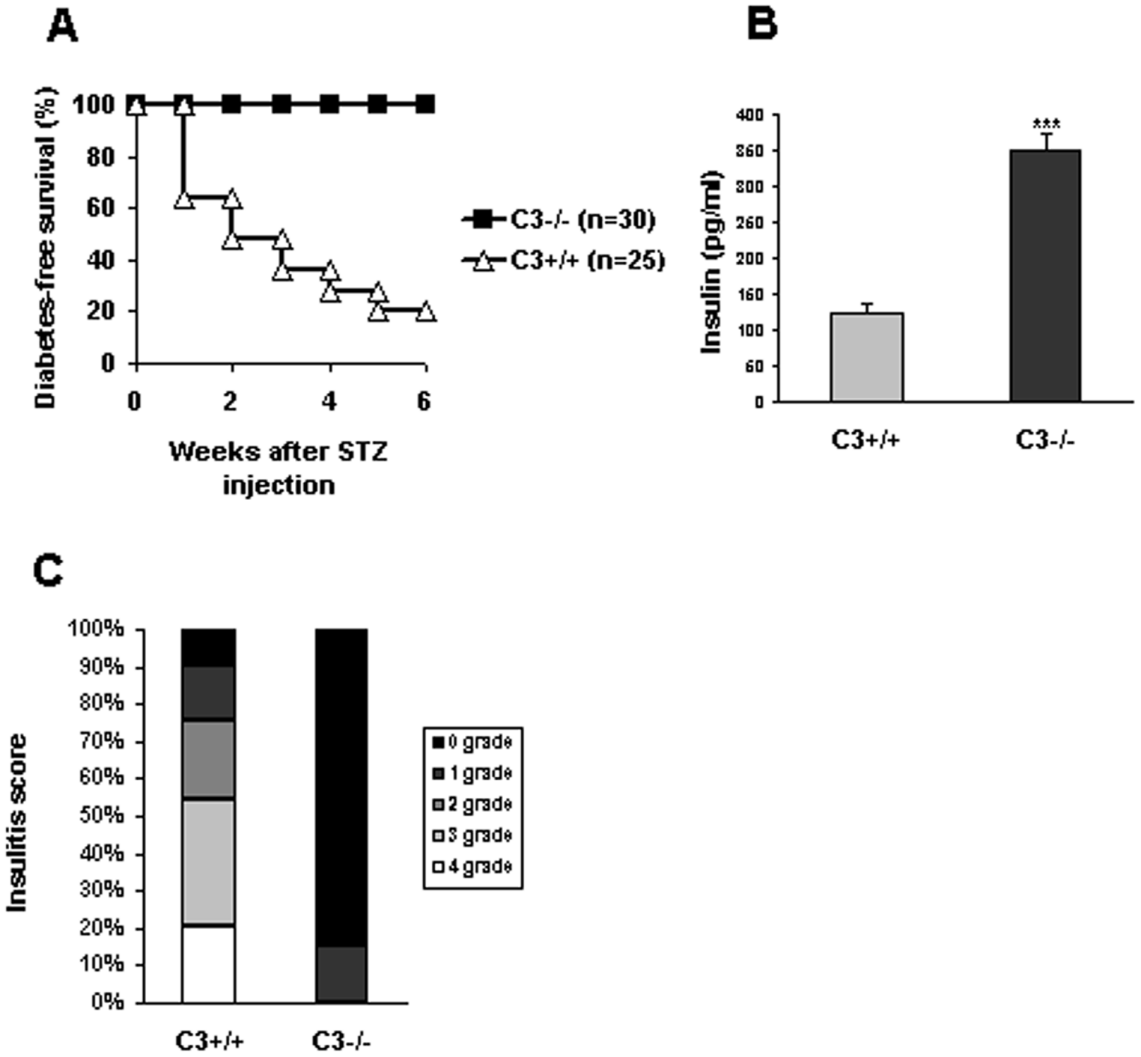
C3 ablation renders resistence to overt diabetes after STZ administration. (**A**) C3−/− mice were injected with STZ at the dose of 40 mg/kg for consecutive 5 days. As controls, C3+/+ mice received the same procedure. Blood glucose was monitored every week and diabetes was diagnosed as ≥300 mg/dl for consecutive 2 weeks. Data were shown as the percentage of diabetes-free mice and representatives of two independent experiments. (**B**) 5 weeks after STZ injection, serum was collected from C3−/− and C3+/+ mice (n = 5) respectively and analyzed for insulin contents by ELISA. Data were shown as mean±SD and representatives of three independent experiments. C, pancreas was isolated, conventionally processed, and stained with H&E. Insulitis scores were performed by an external experienced pathologist. Sixty-eight islets in C3+/+ controls and eight-four islets in C3−/− mice were scored. ***, *P*<0.001 compared with controls.

As described previously [11], STZ-induced autoimmune diabetes was established by repeated injection of low-dose STZ. Blood glucose level was monitored every week. 80% (20/25) of C3+/+ mice were exposed to hyperglycemia by 5^th^ week after STZ administration, while all (30/30) of C3−/− mice were still diabetes-free survival by 6^th^ week (Fig. 1A). In agreement with glucose changes, C3−/− mice had much more amounts of insulin than C3+/+ counterparts (C3−/−: 350.17±22.4 pg/ml vs C3+/+: 123.87±13.45 pg/ml) (Fig. 1B), indicating that C3 ablation remarkably protects insulin-producing cells against autoimmune response-mediated destruction induced by STZ administration.

The effect of C3 deficiency on the progression of insulitis was also examined. The results showed that a total of 85% of the examined islets from STZ-treated C3−/− mice was intact, whereas >50% of the islets from C3+/+ mice showed moderate or severe insulitis by 6^th^ week (Fig. 1C). These data indicate that C3 ablation efficiently prevents autoimmune response-mediated destroy of islets after STZ injection.

### Resistance to STZ-induced Diabetes in C3−/− mice is Associated with Down-regulation of Proinflammatory Cytokine Expression

**Figure 2:**
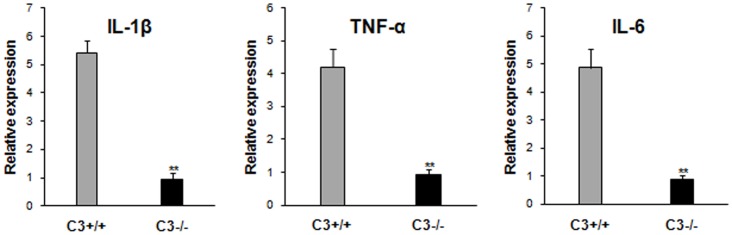
C3 deficiency reduces proinflammatory cytokine expression in the lesions after STZ. Pancreas was dissected from C3+/+ and C3−/− mice 3 weeks afte STZ injection and homogenated. mRNA was isolated from homogenates and IL-1β, TNF-α, IL-6 expression was examined by quantitative RT-PCR. Data represent the mean±SD of five to eight mice per group from two independent experiments. **P<0.01 vs C3+/+ mice after STZ.

To characterize local inflammatory response induced by STZ treatment, the pancreas tissues were dissected and homogenized, then RNA was extracted. We found that the expression of several proinflammatory cytokines including IL-1β, TNF-α, IL-6 was augmented significantly in overt diabetic mice (Fig. 2). C3 ablation led to a considerable down-regulation of their expression (Fig. 2), which may account for the diabetes-resistant effects of C3 deficiency.

### MDSC are Involved in Tolerance of C3−/− Immune Cells to Islet after STZ

**Figure 3:**
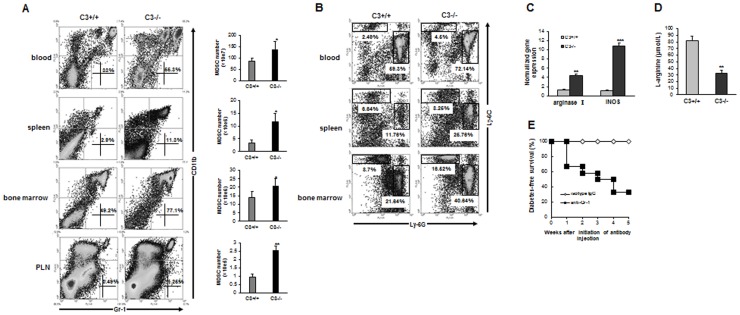
MDSC are indispensable for prevention of STZ-induced diabetes in C3−/− mice. (**A**) cells from whole blood, spleen, bone marrow and pancreatic lymph nodes (PLN) were fractionated 3 weeks after STZ injection from C3+/+ and C3−/− mice and the percentages of Gr-1^+^CD11b^+^ cells were analyzed by flow cytometry. (**B**) at the same timepoint, the percentages of G-MDSC (Ly-6G^hi^Ly-6C^lo^CD11b^+^) and M-MDSC (Ly-6G^−^Ly-6C^hi^CD11b^+^) in the blood, spleen and bone marrow of C3+/+ and C3−/− mice were detected. Representative plots from three independent experiments were shown. (**C**) MDSC from spleen of STZ-treated mice were sorted by FACS. mRNA expression of arginase I and iNOS was examined by qPCR. Accumulative data from four separate experiments were shown. (**D**) L-arginine level in plasma of STZ-treated mice was detected by HPLC. The number is 6 for C3+/+ mice and 10 for C3−/− littermates respectively. (**E**) STZ-treated C3−/− mice without overt diabetes were injected i.p. with anti-Gr-1 antibody (n = 12) or isotype IgG (n = 10) at indicated doses and intervals in Materials and methods. Blood glucose level was monitored every week and the percentage of diabetes-free survival was calculated. **, *p*<0.01; ***, *p*<0.001 compared with C3+/+ counterparts.

Our study has shown that splenic lymphocytes from C3−/− mice display tolerogenic response to islet autoantigens (e.g. GAD and insulin) and Tregs are implicated in this process [11]. To further investigate whether other immune cell populations with immunosuppressive activity contribute to diabetes-free survival of C3−/− mice after STZ, MDSC are focused on in this study as this population is known to be a key regulator for anti-oncogenic response in tumor environment [12], [18]. Notably, the importance of MDSC in prevention from autoimmune diabetes has been underlined recently [17]. First, the percentage of MDSC (Gr-1^+^CD11b^+^) in lymphoid compartment was detected. We found that the abundance of MDSC in the proximal compartment (pancreatic lymph node) and distal compartment (blood, spleen, and bone marrow) of C3−/− mice was significantly higher than those in C3+/+ counterparts 3 weeks after STZ (Fig. 3A). Of note, under steady state, MDSC number in C3−/− mice was comparable to that in C3+/+ littermates (Fig. S1). We also detected the percentage of two subpopulations of MDSC (i.e. granulocytic-MDSC and monocytic-MDSC) respectively. The result showed that STZ treatment led to the expansion of monocytic-MDSC as well as granulocytic-MDSC in C3−/− mice (Fig. 3B). Furthermore, when MDSC from the spleen of STZ-treated mice at the same timepoint, high-level expression of arginase I and iNOS in C3−/− MDSC was observed (Fig. 3C), indicating these two components as important mediators for immunoregulatory function of MDSC. In accord with this, L-arginine contents in the plasma of C3−/− mice were much fewer than C3+/+ counterparts (Fig. 3D). These data suggest the presence of MDSC in C3−/− STZ-treated mice, which may be responsible for diabetes resistance after STZ.

To further investigate the implication of MDSC in diabetes-free survival of C3−/− mice after STZ, MDSC was depleted by anti-Gr-1 administration and the efficacy of depletion was confirmed by flow cytometry (Fig. S2). The results showed that the elimination of MDSC led to occurrence of full-blown diabetes in C3−/− STZ-treated mice (Fig. 3E), underscoring the importance of MDSC for resistance of autoimmune diabetes in C3−/− mice after STZ.

### MDSC Inhibit Effector T Cell Expansion and Diabetogenic Response in Arginase/iNOS-dependent Manner

**Figure 4:**
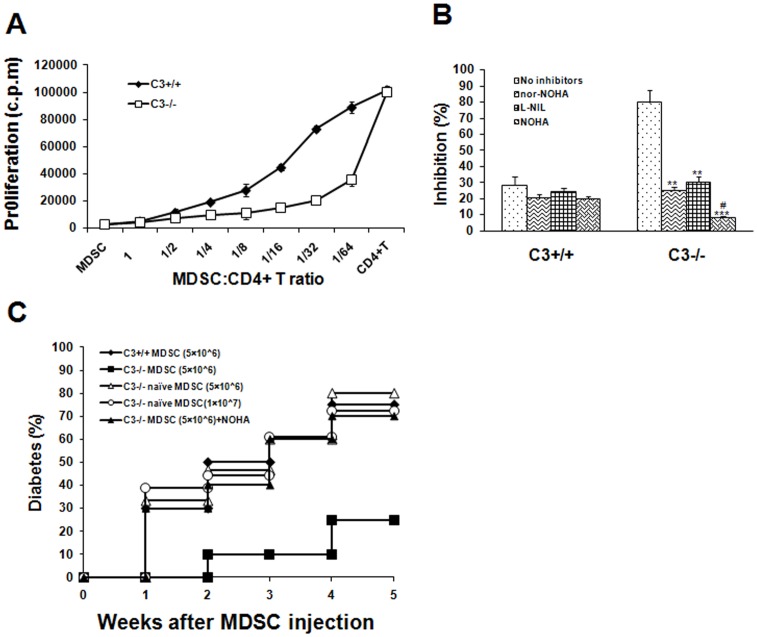
MDSC from STZ-treated C3−/− mice display potent immunosuppressive activity in vitro and in vivo. (**A**) MDSC from the spleen of C3+/+ or C3−/− mice 3 weeks after STZ injection were fractionated by FACS sorting. T-cell depleted spleen cells were irradiated at 3000 rads and used as APCs. Purified CD4^+^ T cells from diabetic WT mice (2×10^5^/well) were cultured with APCs (1×10^5^/well), anti-CD3 mAb (0.5 µg/ml), and in the presense of MDSC at titrated concentration. As controls, MDSC (2×10^5^/well) were cultured alone. Cell proliferation was measured by incorporation of (^3^H)thymidine. Data were shown as mean±SD and representatives of two independent experiments. (**B**) arginase and/or iNOS inhibitors were added into the coculture of MDSC and CD4^+^ T cells at 1∶32 ratio with stimulation of APCs and anti-CD3. Proliferation was examined by incorporation of (^3^H)thymidine. Inhibition rate for coculture of MDSC and CD4^+^ T cells corresponding to CD4^+^ T cells alone was calculated. Data were shown as mean±SD and representatives of three independent experiments with 5–6 mice per group. **, *P*<0.01; ***, *P*<0.001 compared with controls without inhibitors. #, *P*<0.05 compared with those treated with arginase or iNOS inhibitor alone. (**C**) MDSC from the spleen of C3+/+ or C3−/− mice 3 weeks after STZ administration were isolated respectively and injected i.v. (5×10^6^/mouse) into C3+/+ recipients. To evaluate the effect of arginase/iNOS inhibition in vivo, some mice received i.p. administration of NOHA (150mg/kg) simultaneously. Thereafter, STZ-induced diabetes was established in these C3+/+ recipients according to the regimen described above. As controls, MDSC from C3−/− naive mice were infused into C3+/+ recipients followed by STZ treatment at indicated number. Blood glucose was monitored every week. Accumulative data from three experiments were shown. n = 15–20 per group.

To identify the actively suppressive ability of MDSC, the coculture experiment was performed. Gr-1^+^CD11b^+^ cells were purified from the spleen of C3+/+ and C3−/− mice 3^rd^ week after STZ, and cocultured them with CD4^+^ T cells from diabetic WT mice in the presence of immobile anti-CD3 and irradiated APC at a titrated ratio. Gr-1^+^CD11b^+^ cells did not proliferate when cultured alone. Meanwhile, as shown in Fig. 4A, these cells efficiently suppressed proliferation of CD4^+^ effector cells. This suppressive effect was more potent for C3−/− Gr-1^+^CD11b^+^ cells than those from C3+/+ groups, as suppression was routinely observed at a MDSC/CD4^+^ T ratio of <1∶32. These results indicate the presence of a population of potent suppressive MDSC in C3−/− mice, which is responsible for absence of overt diabetes after STZ.

As shown in Fig. 2*B*, MDSC from STZ-treated C3−/− mice expressed high levels of arginase I and iNOS. To determine whether these components were implicated in MDSC-mediated suppressive effects, inhibitors to arginase I and iNOS was added in the coculture of MDSC and CD4^+^ T cells. The results showed that inhibition of activity significantly abrogated suppressive effects of MDSC (Fig. 4B). Moreover, synergetic effects of arginase I and iNOS on mediating immunomodulatory function of MDSC were observed as inhibition of arginase or iNOS activity simultaneously pronouncedly abolished suppressive ability of MDSC compared with those treated with arginase or iNOS inhibitor alone (Fig. 4B), indicating that the suppressive function of MDSC is arginase I and/or iNOS-dependent. In contrast, inhibition of arginase or iNOS activity did not significantly influence the inhibitory function of Gr-1^+^CD11b^+^ cells from STZ-treated C3+/+ mice.

To further examine the diabetes-suppressive function of MDSC in vivo, this population was isolated from the spleen of C3+/+ or C3−/− mice 3 weeks after STZ treatment and infused into C3+/+ recipients followed by STZ. As expected, Gr-1^+^CD11b^+^ cells from STZ-treated C3+/+ mice had no effects on diabetes progression. In contrast, MDSC from STZ-treated C3−/− mice dramatically prevented/delayed the onset of overt diabetes at the number of 5×10^6^ cells/mouse (Fig. 4C), suggesting a protective role of C3−/− MDSC. To address whether Gr-1^+^CD11b^+^ cells from C3−/− naïve mice are immunosuppressive, C3+/+ mice received Gr-1^+^CD11b^+^ cells from C3−/− naïve mice thereafter STZ-induced diabetes was established. The results showed that these cells did not blocked the development of autoimmune diabetes, even if higher number (1×10^7^/mouse) of Gr-1^+^CD11b^+^ cells was infused (Fig. 4C). To investigate whether arginase/iNOS was important for mediating the protective role of MDSC in C3−/− STZ-treated mice, inhibitors to arginase/iNOS were injected into mice receiving C3−/− MDSC. Combined inhibition of arginase/iNOS expression using NOHA almostly reversed the preventive function of C3−/− MDSC on STZ-induced diabetes (Fig. 4C). Similar effects were observed when specific inhibitors to arginase or iNOS were used (data not shown). These data indicate that STZ administration in C3−/− mice render induction of arginase/iNOS-expressing MDSC, which inversely dampen destructive response to insulin-producing cells.

### TGF-β is Crucial for MDSC Induction in C3−/− Mice with Treatment of STZ

**Figure 5:**
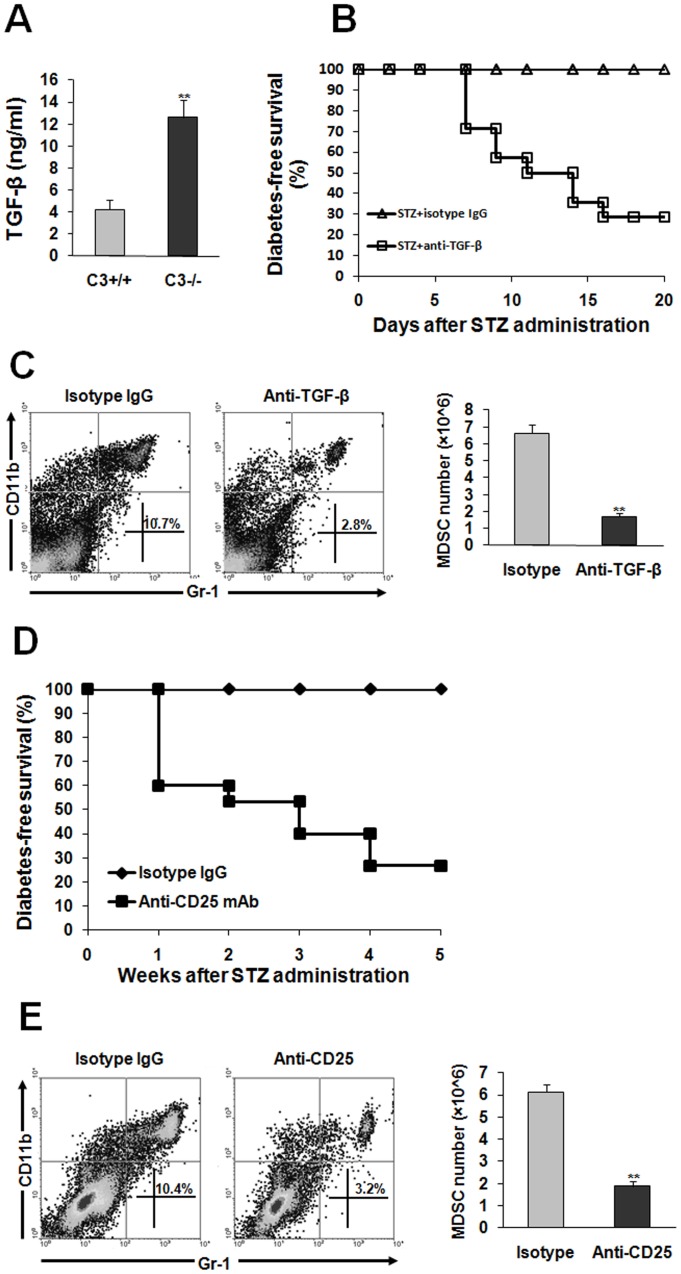
MDSC induction in C3−/− mice subjected to STZ is TGF-β-dependent. (**A**) the plasma from C3+/+ (n = 8) or C3−/− mice (n = 15) was collected 3 weeks after STZ treatment. TGF-β contents were examined by ELISA. (**B,C**) TGF-β antibody or isotype IgG was adminidtrated to STZ-treated C3−/− mice as described in Materials and methods. (**B**) blood glucose was monitored and the percentage of diabetes-free survival was expressed. Data were pooled from three independent experiments. Each group consists of 10–15 mice. (**C**) 4 weeks later, spleen cells were fractionated. The percentage and number of Gr-1^+^CD11b^+^ cells was detected by flow cytometry. Representative data were shown from three separate experiments. (**D,E**) anti-CD25 antibody or isotype IgG was injected into STZ-treated C3−/− mice as described in Materials and methods. (**D**) diabetes-free survival was monitored every week. Each experiment group consisted of 10–15 mice. (**E**) 4 weeks later, the percentage and number of Gr-1^+^CD11b^+^ cells was examined by flow cytometry. Representative data from two separate experiments were shown. Number is 5 to 8 per group when the abundance of MDSC was calculated. **, P<0.01 compared with controls.

Next, we address which factor is critical for appearance of diabetes-suppressive MDSC in C3−/− mice subjected to STZ. As described in our previous study [11], TGF-β has been proposed to be important for maintenance of immune homeostasis in our system. Therefore, we hypothesize that TGF-β is a key factor to induce MDSC thereby inhibit diabetogenic response. Consistent with previous report [11], the plasma of C3−/− mice contained much more contents of TGF-β than C3+/+ mice 3 weeks after STZ administration (Fig. 5A). To identify the role of TGF-β in inducing MDSC, neutralizing antibody to TGF-β was administrated into STZ-treated C3−/− mice. Indeed, neutralization of TGF-β led to occurrence of full-blown diabetes in C3−/− mice after STZ injection (Fig. 5B). More importantly, blockade of TGF-β bioactivity significantly decreased MDSC number in the spleen of STZ-treated C3−/− mice (Fig. 5C), indicating that immunomodulatory function of TGF-β is partially attributed to inducing immunosuppressive MDSC. Given that TGF-β is one of key elements produced by Tregs, we assume that Treg expansion in STZ-treated C3−/− mice may be render induction of MDSC. To address this issue, depletion of Tregs was performed. As our previous report [11], Treg depletion led to the establishment of overt diabetes in C3−/− mice after STZ treatment (Fig. 5D). Strikingly, eliminating Tregs dramatically abolished induction of MDSC (Fig. 5E), which was similar to the results caused by TGF-β neutralization.

## Discussion

In this study, we provide evidence that MDSC as one of regulators of immune responses are intimately implicated in preventive effects of C3 ablation on STZ-induced diabetes. As described in many pathological conditions [12]–[14], these cells highly expressed arginase and iNOS, the latter are important mediators for suppressive ability of MDSC via catalyzing L-arginine into L-ornithine and urea or citrulline and nitric oxide. In accord with this, the contents of L-arginine in plasma of STZ-treated C3−/− mice decreased significantly. As L-arginine is required for T cell receptor expression and antigen-specific proliferation of T cells [19], it is reasonable that arginase/iNOS-expressing MDSC from STZ-treated C3−/− mice hydrolyze L-arginine thereafter inhibit destructive response of T cells to self islets. Indeed, C3−/− MDSC actively suppressed effector T cell proliferation in vitro in arginase/iNOS-dependent fashion. Furthermore, depletion of MDSC or inhibition of arginase/iNOS activity is sufficient to result in the onset of overt diabetes in C3-deficient mice after STZ. Considering several other mechanisms used by MDSC to inhibit pathogenic T cell response including up-regulation of cyclooxigenase-2 and prostaglandin E2 [20], production of TGF-β [21], [22], depletion of cystein [23], and down-regulation of T cell L-selectin expression [24], especially in tumor models, future work is needed to investigate whether one or more mechanisms are utilized by MDSC to inhibit diabetogenic T cell response in our system.

Interestingly, when transfer of STZ-treated C3−/− MDSC into C3+/+ recipients, these cells pronouncedly prevented/delayed the development of diabetes. Transfer of equal or even more number of Gr-1^+^CD11b^+^ cells from C3−/− naïve mice, however, has no effects. Consistent with this, Gr-1^+^CD11b^+^ cells from C3−/− naïve mice express arginase/iNOS at very low level. These observations indicate that under steady state C3 deficiency is not sufficient to induce MDSC. When STZ is administrated, autoantigens from islet cells are released, which may facilitate the generation of MDSC.

In terms of the mechanisms responsible for MDSC induction in C3−/− mice after STZ, we proposed a critical role of TGF-β in inducing MDSC, as blockade of TGF-β bioactivity abolished induction of MDSC in STZ-treated C3−/− mice. Moreover, depletion of Tregs also led to failure of MDSC induction and establishment of full-blown autoimmunity. Thus, on the basis of the data from our previous study [11] and the current report, it is plausible that, when STZ was administrated, islet autoantigens were released, which favor C3−/− DC actively prompt TGF-β-secreting Treg expansion. Thereafter, Tregs promote induction of MDSC, the latter inhibit pathogenic response of effector T cells to self islets. Several studies have shown that MDSC have ability to induce Tregs through producing TGF-β [25], [26], so this possibility can not be excluded, considering the fact that TGF-β expression in MDSC from STZ-treated C3−/− mice really up-regulated (data not shown). The capacity of MDSC to promote Treg expansion in our system is being examined. On the other hand, the details in TGF-β inducing the generation of MDSC need to be further investigated. In view of a series of factors involved in inducing MDSC [27]–[29], such as GM-CSF, PGE2, IL-6 etc., whether one or more factors are required for inducing MDSC in this model is worthy to be studied. Overall, the present study has clearly identified Tregs and MDSC as key regulators for resistance to STZ-induced autoimmune diabetes in absence of complement C3.

The nonobese diabetic (NOD) model of diabetes, as one of the primary models for human disease [30], is being used to evaluate the influence of complement deficiency on the development of disease. In pilot study, complement activation in local tissues was observed and administration of complement activation inhibitor FUT-175 exhibited the preventive effects (data not shown). On the other hand, our study underlines the competence of MDSC in dampening autoimmune response and highlights MDSC-based cellular therapy in patients with autoimmune diseases.

## Supporting Information

Figure S1 **C3 deficiency does not affect MDSC number under steady state.** Splenocytes from naive C3+/+ and C3−/− mice were fractionated and the percentage of MDSC was detected by flow cytometry. The plots as representatives of three independent experiments were shown.
(TIF)

Figure S2 **The efficacy of depletion of MDSC by anti-Gr-1 treatment.** 2 weeks after anti-Gr-1 antibody or isotype administration, Splenocytes were fractionated and the percentage of MDSC was detected by flow cytometry. The plots as representatives of two independent experiments were shown.
(TIF)

Figure S3 **Blood glucose level of mice subjected to administration of Gr-1 antibody or isotypes.** STZ-treated C3−/− mice without overt diabetes received anti-Gr-1 antibody or isotypes as described in Materials and methods. Blood glucose level was monitored every week. Representative data of glucose concentration from individuals treated with anti-Gr-1 (n = 5, red line) or isotypes (n = 4, blue line) were shown.
(TIF)
