# Supplementary figures and images for: Resistance to Streptozotocin-Induced Autoimmune Diabetes in Absence of Complement C3: Myeloid-Derived Suppressor Cells Play a Role
Source: PLoS One. 2013 Jun 18;8(6):e66334. doi: 10.1371/journal.pone.0066334 (PMC3688892; doi:10.1371/journal.pone.0066334)

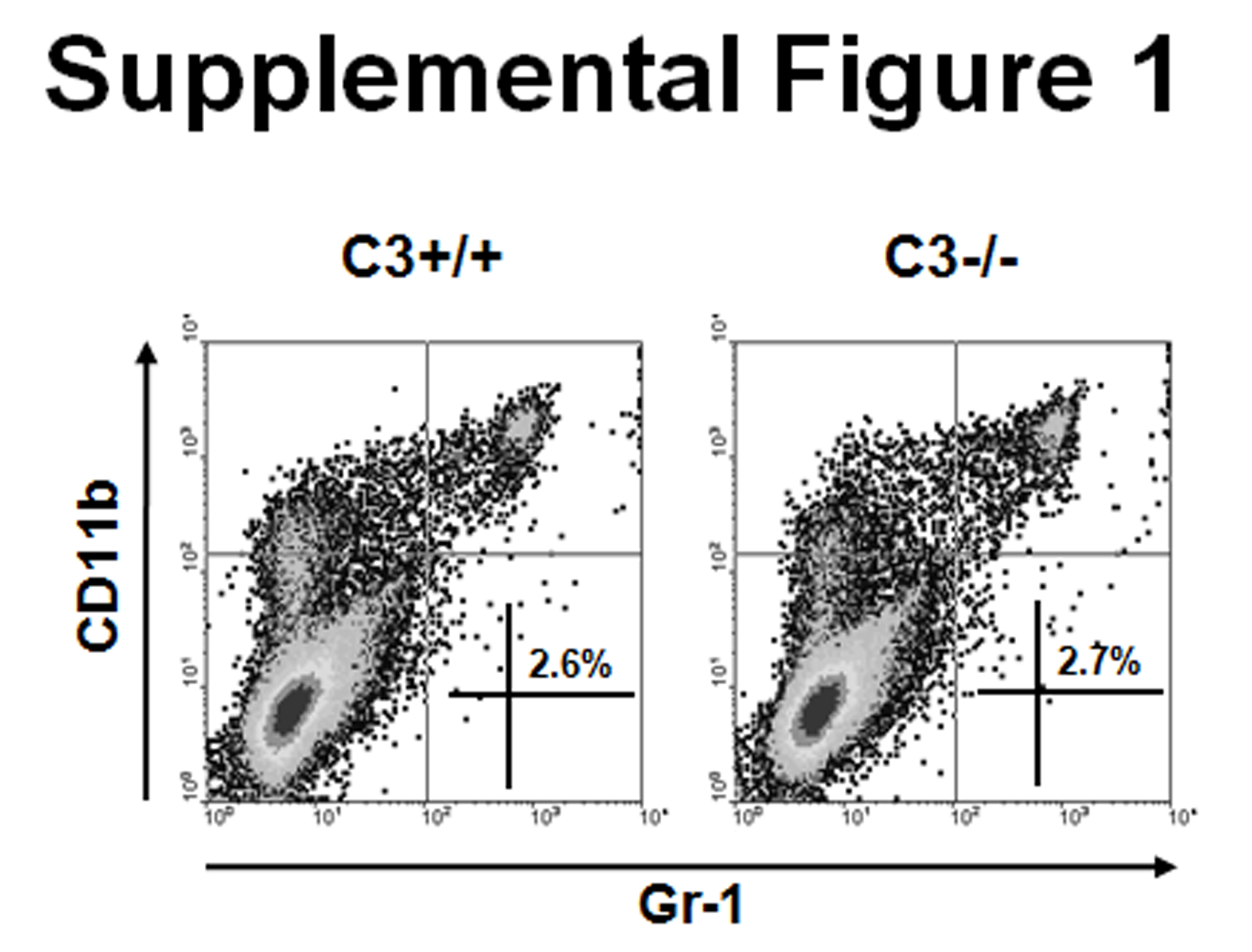

Supplement: Figure S1 — C3 deficiency does not affect MDSC number under steady state. Splenocytes from naive C3+/+ and C3−/− mice were fractionated and the percentage of MDSC was detected by flow cytometry. The plots as representatives of three independent experiments were shown. (TIF) [file pone.0066334.s001.tif]

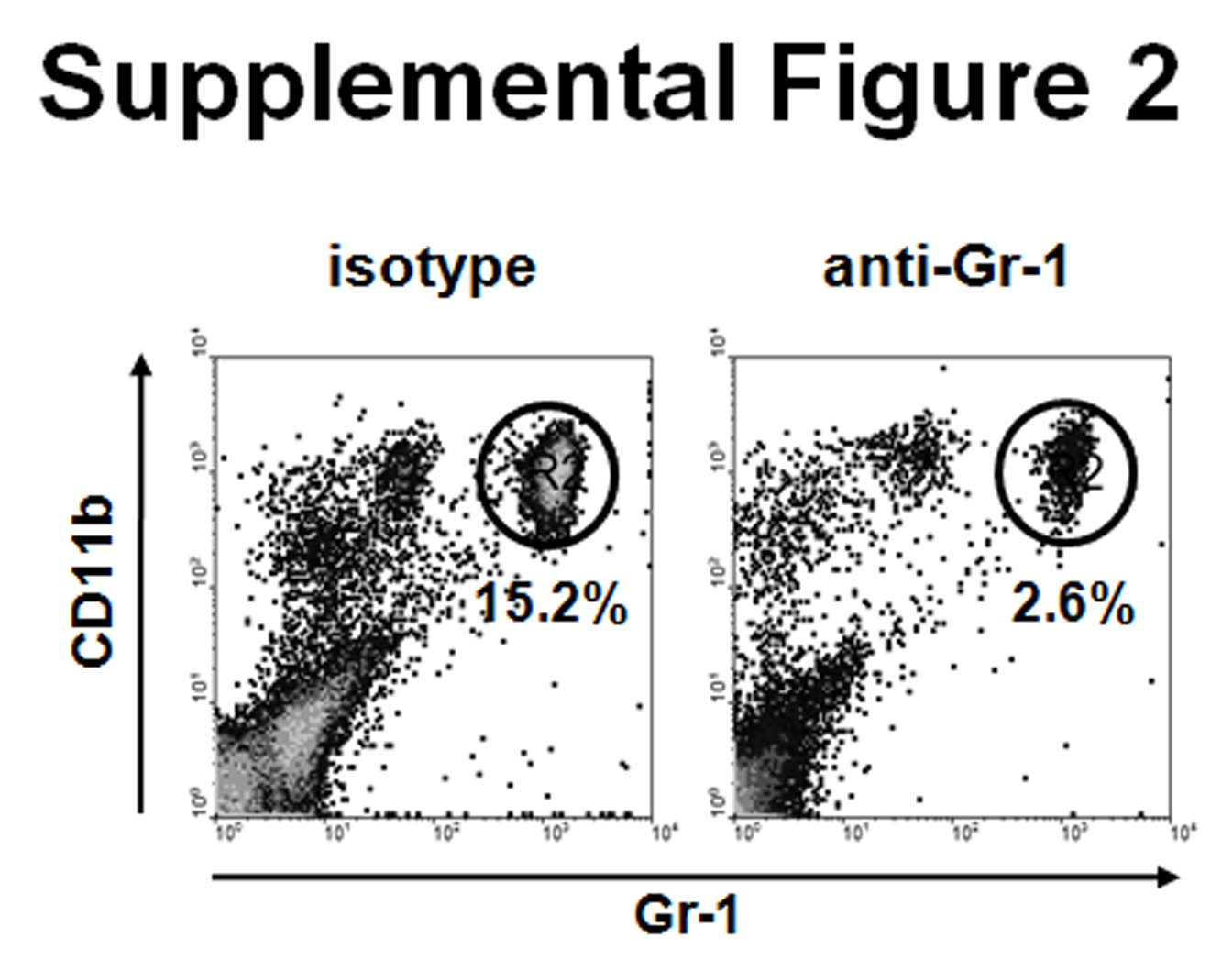

Supplement: Figure S2 — The efficacy of depletion of MDSC by anti-Gr-1 treatment. 2 weeks after anti-Gr-1 antibody or isotype administration, Splenocytes were fractionated and the percentage of MDSC was detected by flow cytometry. The plots as representatives of two independent experiments were shown. (TIF) [file pone.0066334.s002.tif]

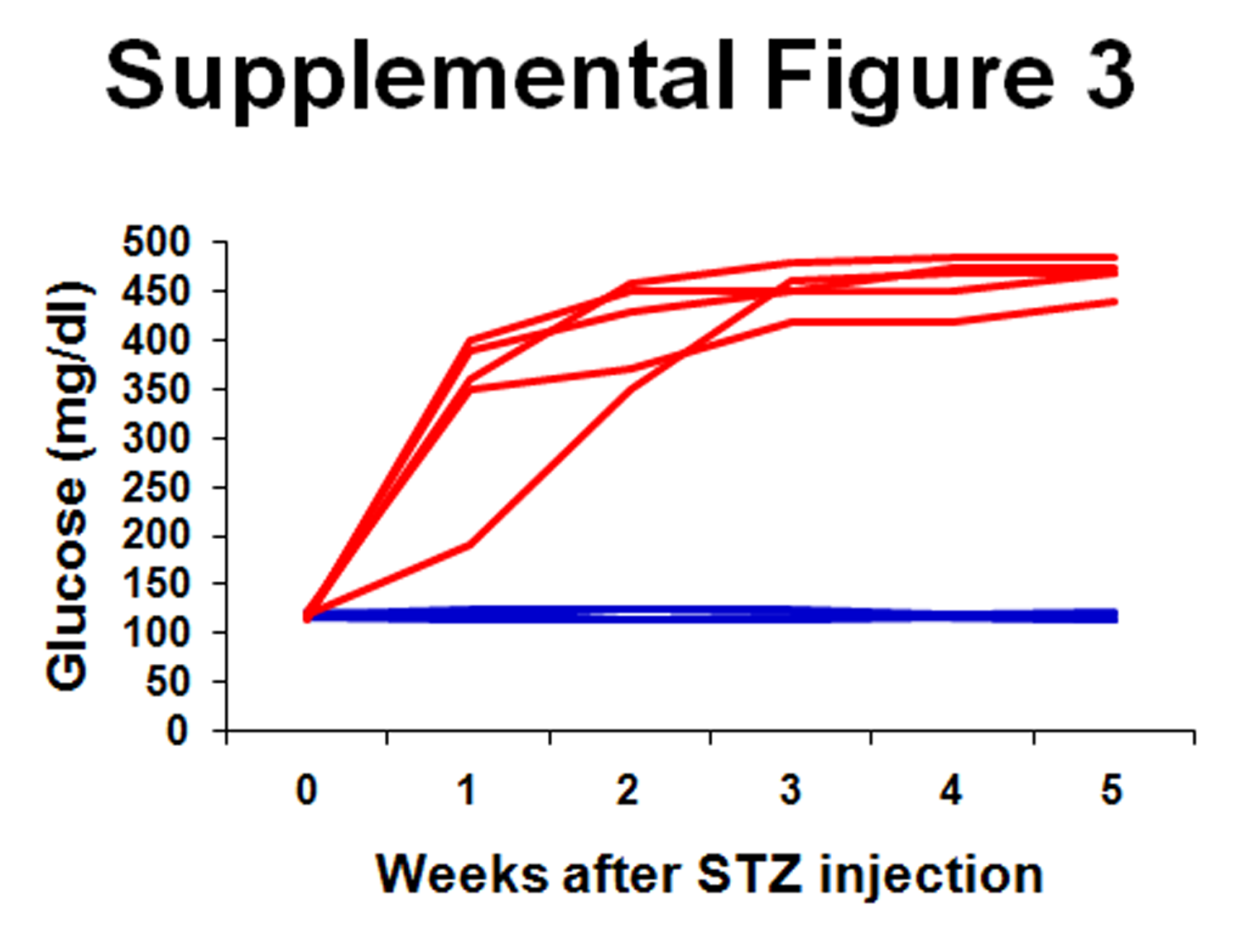

Supplement: Figure S3 — Blood glucose level of mice subjected to administration of Gr-1 antibody or isotypes. STZ-treated C3−/− mice without overt diabetes received anti-Gr-1 antibody or isotypes as described in Materials and methods. Blood glucose level was monitored every week. Representative data of glucose concentration from individuals treated with anti-Gr-1 (n = 5, red line) or isotypes (n = 4, blue line) were shown. (TIF) [file pone.0066334.s003.tif]
